# Establishment and Industrialization of a New Treatment Method Using Regenerative Cardiomyocyte Transplantation for Refractory Severe Heart Failure - Secondary Publication

**DOI:** 10.31662/jmaj.2023-0075

**Published:** 2023-10-05

**Authors:** Keiichi Fukuda

**Affiliations:** 1 Department of Cardiology, Keio University School of Medicine, Tokyo, Japan

**Keywords:** regenerative medicine, heart failure, cell transplantation, iPS cells

## Abstract

Cardiomyocytes undergo cell division during the fetal period but do not divide after birth; thus, they grow into adult heart cells by enlarging their size. Therefore, heart failure occurs when a certain number of cardiomyocytes are lost owing to myocardial infarction, myocarditis, sarcoidosis, etc.

Through scientific efforts, we have developed methods to safely and efficiently generate induced pluripotent stem (iPS) cells from peripheral blood T cells, generate ventricle-specific cardiomyocytes from iPS cells, and remove residual iPS cells and non-cardiomyocytes using the “metabolic selection method” and purify the cardiomyocytes from iPS cell derivatives. We have also developed the technology to mass-produce and efficiently engraft cardiomyocytes by generating cardiomyocyte spheroids and have developed devices suitable for cell transplantation.

We have confirmed the safety and efficacy of these techniques by performing preclinical studies (oncogenesis, arrhythmogenicity, etc.) using immunodeficient mice, rats, pigs, and monkeys. Based on these technologies, we have successfully regenerated human ventricular muscle-specific cardiomyocytes with purity greater than 99%. We have also confirmed that the regenerated myocardium transplanted into immunodeficient mice maintained autonomic beating for more than a year without tumor formation.

We are planning to conduct clinical trials to transplant iPS cell-derived cardiomyocytes into patients with heart failure associated with ischemic heart disease, which will, in the near future, enable clinical applications using HLA-deficient iPS cells and iPS cells generated from the patient’s own lymphocytes to generate regenerative cardiomyocytes without rejection. It would also help establish personalized medicine for heart failure and usher in the long-awaited treatment for intractable severe heart failure using ventricular muscle supplementation.

## Introduction

Despite the decrease in the early mortality rate for heart failure owing to improvements such as acute-phase catheter treatment for myocardial infarction and the development of effective oral heart failure treatment drugs, the number of patients with chronic heart failure has increased over the past 30 years ^(1)^. This rate is expected to increase at least until 2050 and has been named “the heart failure pandemic” ^(2)^. The left ventricle consists of approximately 4 billion ventricular myocytes ^(1)^. During myocardial infarction, myocarditis, cardiomyopathy, and similar ailments, the number of cardiomyocytes decreases owing to necrosis. As cardiomyocytes do not regenerate, the heart attempts to compensate for the function through hypertrophy of the remaining cardiomyocytes; however, incomplete compensation leads to heart failure. To date, heart failure has been treated by modifying neurohumoral factors to improve the function of the remaining myocardium ^(3), (4)^. However, in this newly developed method, heart failure is treated by compensating for the cardiomyocyte shortage through transplantation. We have developed a method for generating ventricular muscle-specific cardiomyocytes from human induced pluripotent stem (iPS) cells, which are purified to a high degree before being transplanted into the left ventricular wall with high efficiency. In this paper, the developmental process has been discussed, along with its future prospects in the treatment of heart failure.

## Production of Cardiomyocytes Suitable for Regenerative Medicine

Ever since Professor Shinya Yamanaka created human iPS cells ^(5), (6)^, many researchers have attempted to create cardiomyocytes using iPS cells as a better alternative to using embryonic stem (ES) cells. The different types of cardiomyocytes include the sinus node, atrial muscle, ventricular muscle, and Purkinje cells; they differ greatly in cell morphology, ion channels, contractile proteins, and electrophysiological and physiological characteristics. Ventricle-specific cardiomyocytes are required for the treatment of heart failure because they are transplanted into the left ventricular wall. We have been using human ES cells ^(7)^ since 2001 and human iPS cells since 2007 to develop a cardiomyocyte-differentiation-induction technology. At present, this technology has been improved to enable the selective differentiation of atrial and ventricular muscles, particularly as ventricle-specific cardiomyocytes are produced and used in regenerative medicine. In fact, at the time of transplantation, part of the cardiomyocyte phenotype remains in the fetal ventricular stage, although the transplanted ventricular muscle is a complete adult-type ventricular muscle. In recent years, novel methods have been developed to selectively induce the differentiation of sinus node (pacemaker) cells ^(8)^. Hopefully, in the near future, cardiomyocytes can be selectively used according to their function.

## Prevention of Tumorigenesis, a Critical Issue in iPS Cells

**Figure 1.**
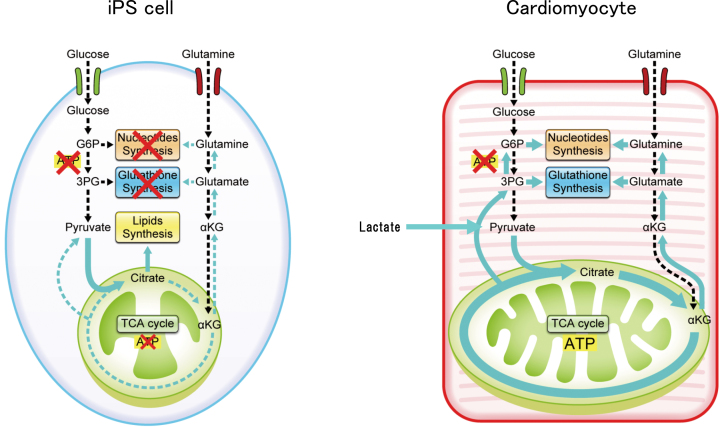
Differences in energy metabolism between iPS cells and cardiomyocytes. Although iPS cells use both glucose and glutamine as their main energy sources, cardiomyocytes additionally use lactate as an energy source. In a culture medium lacking glucose and glutamine but containing lactic acid, only cardiomyocytes can survive, whereas undifferentiated iPS cells and non-cardiomyocytes cannot. Thus, cardiomyocytes can be purified.

iPS cells have a high growth rate and pluripotency, which make them highly suited as materials in regenerative medicine. However, not all cells can differentiate into the target cells. The remaining nontarget cells and a certain number of undifferentiated iPS cells form a tumor called teratoma, which hinders clinical application. Therefore, a purification step is required. To solve this problem, we performed a detailed analysis of the energy metabolism-related pathways of cardiomyocytes and iPS cells and landed on a new solution. As iPS cells have a higher cell proliferative capacity than that of cardiomyocytes, the respective energy metabolism pathways are significantly different. In iPS cells, the high proliferation rate is maintained by the consumption of large amounts of glucose and glutamine. Pyruvate, the final metabolite of the glycolytic pathway, is converted into fatty acid and lactic acid without being consumed in the mitochondria; the fatty acids are used for cell membrane formation, whereas lactic acid is excreted from the cell (Figure 1). However, the cardiomyocytes utilize glucose and glutamine and actively uptake lactate. Lactate is converted to pyruvate by lactose dehydrogenase, which is utilized by the developed mitochondria to synthesize a large amount of ATP through the tricarboxylic acid cycle. Based on these findings, we have developed a method to eliminate iPS cells and non-cardiomyocytes by removing glucose and glutamine and adding lactate to the culture medium. As a result, the cardiomyocytes remain active (continue to beat) but the undifferentiated iPS cell count is reduced to 0.001% (detection sensitivity) or less; the non-cardiomyocyte population is similarly reduced ^(9), (10)^. We also confirmed that tumorigenesis leading to the formation of teratoma did not occur when the purified cells were transplanted into immunodeficient mice.

## Efficient Transplantation Method Using Aggregated Cardiomyocytes

Many studies have used isolated floating cardiomyocytes after freezing and thawing for transplantation ^(11), (12)^. This method involves treatment with trypsin, which leads to the degradation of the isolated cells, cell-surface cell-adhesion molecules, and various growth factor receptors, ion channels, and other cell-surface molecules. Moreover, freezing the cardiomyocytes affects the cell engraftment rate after transplantation, reducing it to less than 5%. A low cell engraftment rate leads to the infiltration of inflammatory cells to remove the necrotic cells, causing inflammation. Hence, this is not a preferred transplantation method.

**Figure 2.**
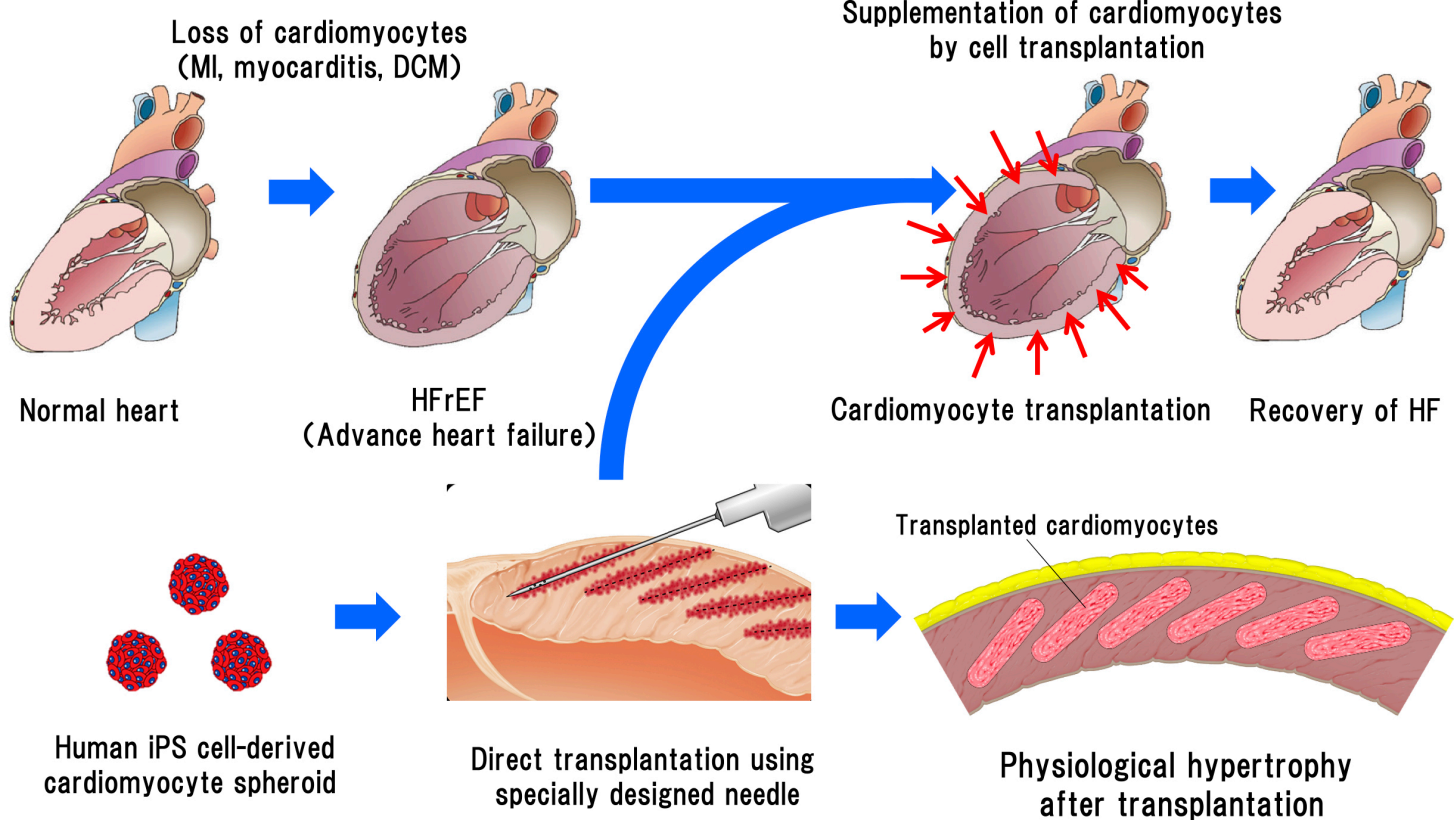
Schematic diagram of regenerative cardiomyocyte transplantation. The engraftment rate of post-transplantation cardiomyocytes was significantly improved by directly transplanting human iPS cell-derived regenerated ventricular cardiomyocyte clusters (cardiomyocyte spheroid) into the left ventricular wall using a special transplantation needle.

As transplantation of sheet-like tissue onto the epicardial surface cannot directly contact the host myocardium owing to the interference of the epicardium and epicardial adipose tissue, its beating is not synchronous with that of the host myocardium ^(13)^. To solve this problem, we need a new method where transplantation can be performed without trypsin treatment, the cells can adhere directly to the host myocardium and form an electrical connection, and angiogenesis is induced. The method we have developed involves the formation of minute aggregates of cardiomyocytes called cardiomyocyte spheroids. A cardiomyocyte spheroid is an aggregate of approximately 1,000 cardiomyocytes. We collaborated with a company to produce a specially designed micro-well plate to produce these spheroids. The advantages of using cardiomyocyte spheroids are that the cells can be harvested without trypsinization, the cell-surface proteins are not damaged, the extracellular matrix is retained, and humoral factors such as cell growth factors secreted from cardiomyocytes are intact. The transplantation of cardiomyocyte spheroids is comparable to that of micro-heart tissue (Figure 2); hence, the cell-survival rate after transplantation is remarkably increased ^(14)^. As the size of iPS cells is small and approximately the same as that of white blood cells, the transplanted regenerated cardiomyocytes are very small at the beginning of transplantation; however, they exhibit gradual growth and physiological hypertrophy post transplantation. The morphological changes in the cardiomyocytes also occur gradually post transplantation to form an elongated shape in the longitudinal direction, similar to that of adult cardiomyocytes ^(15)^. No tumor formation, such as teratoma, was observed a year after transplantation.

## Development of Implantable Devices

We observed that intracoronary administration of cardiomyocyte spheroids engrafted only a small portion of the cells, but most of the cells were not retained in the myocardium and caused myocardial infarction at the site of the cardiomyocyte spheroid-injected site of the coronary artery of the heart. Therefore, we concluded that intracoronary administration was not a suitable transplantation method.

Thus, we needed to develop a transplantation device that would allow the regenerating myocardium to survive safely and stably for a long period. We first developed a specially designed less-invasive needle for transplanting the cardiomyocyte spheroids from the epicardial heart surface under thoracotomy. The blade tip of a normal injection needle enables easy insertion into the tissue but it cuts the coronary microvessels, which causes bleeding that interferes with transplantation. Therefore, we developed an injection needle with a cone-shaped blind end without a blade at the tip, resembling acupuncture needles. This injection needle has six holes on the side to allow the injection of cardiomyocyte spheroids into the heart tissue, and cell transplantation has been performed with almost no bleeding ^(16)^. As a result of these innovations, the transplanted cardiomyocytes were found to engraft efficiently in heart tissue for a long period. The transplanted cardiomyocytes were observed to be very small in size immediately after transplantation, but over time, they enlarged physiologically and began to morphologically resemble the adult myocardium by elongating in the longitudinal direction. The transplanted cells did not develop tumors such as teratomas. In addition, vascular endothelial cells migrated from the surrounding myocardial tissue into the transplanted myocardium, forming a capillary network over time and providing blood flow.

## Can Arrhythmias Reported during Cardiomyocyte Transplantation Be Overcome?

Previous studies that regenerated cardiomyocytes by transplanting human ES or monkey iPS cells into monkey hearts reported that sustained ventricular tachycardia was observed for approximately 1 month after transplantation ^(11), (12), (17)^. This is regarded as a major issue in cardiac regenerative medicine. They suggested that engraftment arrhythmia induced by transplantation of human cardiomyocytes is transient and that pharmacological therapy can effectively suppress these arrhtymias ^(18)^. In these studies, cardiomyocytes were transplanted immediately after thawing the frozen cells, resulting in a low engraftment rate (approximately 5%). In contrast, when we transplanted human iPS cell-derived regenerative cardiomyocytes into five monkey hearts, we observed ventricular tachycardia only in one animal for 9 min. We attributed the reason for this difference to our transplantation method, which is characterized by (1) specificity to the cardiomyocytes of the ventricular muscle, enabling a low spontaneous beating rate (low automaticity); (2) high purity of the cardiomyocytes and absence of non-cardiomyocyte clumps; (3) negligible inflammation associated with the treatment of necrotic cells; and (4) negligible vascular damage owing to the small number of cells transplanted at each site; hence, ischemia is less likely to occur around the transplant site.

## Future Prospects

Research in regenerative medicine has been showing steady progress. Cardiac regenerative medicine faces many hurdles in developing the ideal differentiation induction method, purification of cardiomyocytes, elimination of iPS cells, transplantation methods, and transplantation devices. However, these difficult problems have been overcome by constant technical innovation. The universal expectation is for a transplantation intervention that does not require a donor. This depends on the successful industrialization of these innovations. Compared with that in the United States, developing bio-ventures in Japan has been more challenging in the past. However, in recent years, this scenario is changing because of the governmental efforts to nurture academia-initiated ventures. Hence, regenerative medicine of the heart can be incorporated into heart failure treatment clinically by adopting a systematic step-by-step approach to solving the problems that might arise.

## Article Information

This article is based on the study, which received the Medical Award of The Japan Medical Association in 2022. This is a revised English version of the article originally published in Japanese in the Journal of the Japan Medical Association 2023;151(10):1829-32 ^(19)^. The original version is available at https://www.med.or.jp/cme/jjma/newmag/15110/15110.html. Only members of the Japan Medical Association are able to access it.

### Conflicts of Interest

Keiichi Fukuda is CEO of Heartseed Inc.
